# First Evidence for Two Independent and Different Leishmaniasis Transmission Foci in Sri Lanka: Recent Introduction or Long-Term Existence?

**DOI:** 10.1155/2019/6475939

**Published:** 2019-07-25

**Authors:** Yamuna Siriwardana, Bhagya Deepachandi, Shreenika de S. Weliange, Chandanie Udagedara, Chandanie Wickremarathne, Wipula Warnasuriya, Ranthilaka R. Ranawaka, Indira Kahawita, P. H. Chandrawansa, Nadira D. Karunaweera

**Affiliations:** ^1^Department of Parasitology, Faculty of Medicine, University of Colombo, Colombo 00800, Sri Lanka; ^2^Department of Community Medicine, Faculty of Medicine, University of Colombo, Colombo 00800, Sri Lanka; ^3^Teaching Hospital, Kandy 20000, Sri Lanka; ^4^National Hospital, Colombo 00800, Sri Lanka; ^5^Teaching Hospital, Kurunegala 60000, Sri Lanka; ^6^Teaching Hospital, Kalutara 12000, Sri Lanka; ^7^Base Hospital, Homagama 10200, Sri Lanka; ^8^332/4, Anagarika Dharmapala Mawatha, Nupe, Matara 81000, Sri Lanka

## Abstract

Cutaneous leishmaniasis caused by a genetic variant of* L. donovani *is being reported from Sri Lanka since year 2001. Patients presented from different geographical locations (600 patients from North or South and a minority of cases from other foci, 2001-2013) were studied. Analysis revealed two different sociodemographic and clinical profiles of leishmaniasis in Northern and Southern Sri Lanka. Also, the same different profiles were present in these foci since the onset of the recent outbreak and had independently propagated within each focus over the time. A profile of 14 parameters identified in the Northern focus was further examined with regard to other locations. Northwestern (10/14) and Central parts (9/14) of the island were more similar to Northern focus (14/14). Infection would have originated in one focus and spread to other 2 in Northern Sri Lanka. Southern focus was different from and appeared older than all others (2/14). Western focus that accommodates a large transient population had a mixed picture of North and South features (4/14). Lesions in North showed a slow progression and a nonulcerative nature (128/185, 69.2%), while those in South showed a rapid progression and less nonulcerative lesions (193/415, 46.5%). Clinical analysis favoured a parasite aetiology (considerable strain differences) rather than a host aetiology (age, gender, or genetics). Both foci demonstrated a biannual seasonal variation since the onset of the epidemic. Two peaks were observed during the early and latter parts of the year. Furthermore, long-term existence and recent spatiotemporal expansion and detection of leishmaniasis in this country rather than a recent introduction and establishment were indicated by these findings. Vigorous antimalarial activities that existed in Sri Lanka until few decades ago, lack of professional awareness, and more recent military activities that brought human population in close contact with a sylvatic cycle would have played a role in silent propagation of* Leishmania* parasites and subsequent increment in human cases, respectively, in this country.

## 1. Introduction

Many countries in the tropics and subtropics are affected by leishmaniasis with an annually increasing disease incidence [1, 2]. Annual incidence of VL in the developing countries has been estimated as 200* *000–400* *000 cases [3] while new disease foci are also reported continuously [4]. Multiple countries in the Indian subcontinent (ISC) are affected by leishmaniasis since long.* L. donovani *which is considered as the most dangerous and visceralizing parasite species results in potentially fatal visceral leishmaniasis (VL) in this region.

Sri Lanka is a biodiversity hotspot and an equatorial island of 65,610 Km^2^, situated in the Indian Ocean and only 12 Km away from India. However, leishmaniasis was almost unheard of except for few case reports until year 2001 in this country [5, 6]. Increased case numbers were reported after year 2001, probably due to the multiple awareness programs carried out by us immediately following the detection of a recent case from Northern Sri Lanka [7]. A genetic variant of* L. donovani* was subsequently identified as the cause of cutaneous leishmaniasis (CL) in this focus [8, 9]. Meanwhile, further awareness activities in Southern Sri Lanka also resulted in high case reporting from these areas in a similar manner. Local transmission of CL was first reported in 1992 from Southern Sri Lanka [6]. Posing a threat to the ongoing VL elimination efforts in the ISC, a large number of cases are being reported in Sri Lanka at present [10]. In spite of the presence of* L. donovani*, majority of infections seem to remain confined to the skin. Historical evidence also supports existence of both cutaneous and visceral leishmaniasis in Sri Lanka even 100 years ago [11–13].

It could be assumed that the disease was introduced long ago but remained silent due to unawareness, lack of suspicion, and low disease incidence secondary to regular and island wide usage of insecticides to control malaria. Further supporting arguments were provided by genetic studies that demonstrated a local cluster of* L. donovani *that are closely related but distinct from* L. donovani* of the ISC [9, 14, 15]. Clinicoepidemiological studies that identified established biannual seasonal variation in case reporting coinciding with monsoonal rainfall patterns since long also indicate long-term existence of this infection in Sri Lanka [16]. Therefore, it is difficult to comment on original spatiotemporal locations of* Leishmania *parasite introduction to this island.

Long-term existence of infection can also lead to independent evolution of the parasite at different locations resulting in strain variation and the eventual phenotypic, drug response, or epidemiological patterns as well. Supporting this argument, regional variation in risk factors was first suggested in year 2010 [17]. This study identified zoonotic transmission in Northern Sri Lanka and peridomestic transmission in Southern Sri Lanka. More recent studies have further strengthened this finding [18, 19]. In the recent past, minor changing patterns within a still undisturbed main profile of CL [16], detection of visceralized infection in few cases [20, 21], CL associated humoral response [22], widening of spatial distribution of case reporting [16], and evidence for a different entity of atypical skin lesions [23] were also reported. In spite of the spatial expansion of leishmaniasis over time, main case reporting areas still remain confined to Northern and Southern disease foci [16]. It is uncertain what other features show regional variation. Parasite virulence is known to change during progression of an epidemic [24, 25]. Therefore, it is important to examine possibility of intracountry variations of genetic structure, genomic makeup, and other characteristics.

Such findings will be useful in designing disease control strategies locally as well as in the regional drive for leishmaniasis control in ISC that predicts continued* L. donovani* transmission in the region even after 2020 [26]. Current analysis was carried out to describe the regional variation of clinicoepidemiological characteristics of leishmaniasis occurring in Sri Lanka.

## 2. Materials and Methods

Patients with clinical CL presented from different geographical locations were investigated in Faculty of Medicine in University of Colombo (2001 -2014). All patients were recruited after informed consent. Clinical evaluation and data collection was carried out by the principal author or medically qualified persons after training on data collection by the same person (principal author). Laboratory confirmation was done by light microscopy, culture, or polymerase chain reaction (PCR) performed on lesion aspirations, slit scrapings, or punch biopsies [27, 28]. Laboratory confirmed cases were included in further analysis. Lesion data were collected from a randomly selected lesion in case of patients with multiple lesions. Missing and doubtful information was excluded case wise or lesion wise. Working definitions were developed.

### 2.1. Working Definitions

*Primary lesion*: skin nodule measuring ≤1 cm at maximum diameter.

*Size*: maximum diameter of the observable lesion measured to the closest centimetre excluding induration.

*Stages of the epidemic*: early stage: 2001-2003, mid stage: 2004-2008, late stage: 2009-2013. Total study period: all the years from 2001 to 2013.

**Figure 1:**
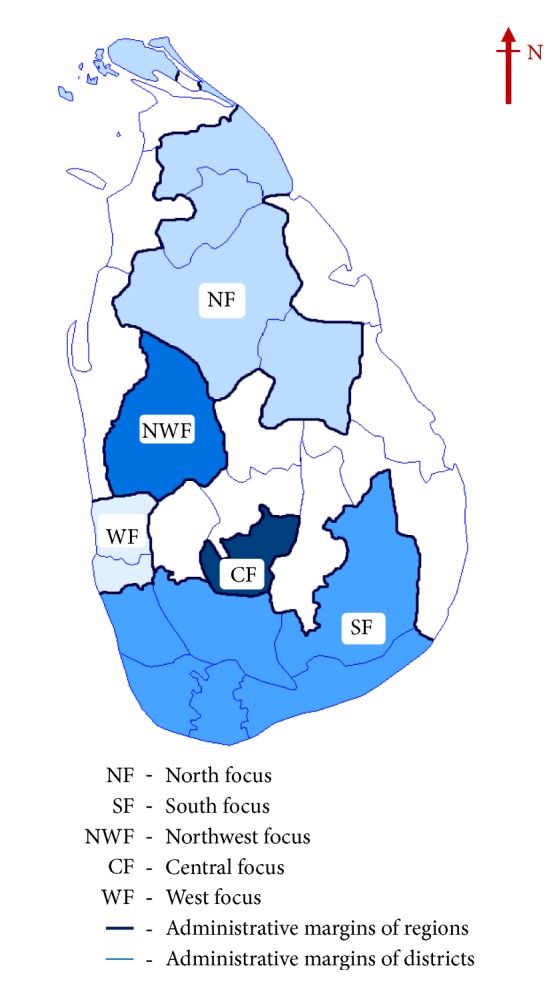
Map of Sri Lanka showing different geographical regions considered in the study. North focus (NF), South focus (SF), Northwest focus (NWF), Central focus (CF), and West focus (WF) represent the different transmission foci within the country.

*Study sites* (Figure 1)

*Main transmission foci* (as identified in Siriwardana* et al*. 2019a [16]): North focus (NF) = Anuradhapura, Jaffna, Mulaitiv, Polonnaruwa, and Vavuniya districts (districts marked as NF in the country map). South focus (SF) =Galle Hambantota, Kalutara, Matara, Moneragala, and Ratnapura districts (districts marked as SF in the country map).

*Other transmission foci*: Northwest focus (NWF) = Kurunegala (district marked as NWF in the country map). Central focus (CF) = Nuwara Eliya (district marked as CF in the country map). West focus (WF) = Colombo and Gampaha (districts marked as WF in the country map).

Northern and Southern transmission foci largely included parts of the dry zones in Sri Lanka.

A sample of 600 (first 200 consecutive laboratory confirmed cases from the early, mid, and late stages of the epidemic) was identified from main disease foci (South focus and North focus). A subgroup of cases reported from other disease foci was also included in the analysis (n=177). Main sociodemographic and clinical features between North and South were compared by descriptive analysis and crosstabulations. Potential aetiological factors were examined using logistic regression modelling and descriptive analysis. Color-coded graphical presentation of 14 selected features was used for comparison between all sites.

### 2.2. Statistical Methods

Data analysis was carried out using SPSS-20 package. Trends in sociodemographic characteristics and clinical features and the duration of the lesion were described using descriptive statistics such as number and proportions for categorical data and mean and standard deviation for continuous data. Relationships of the lesion characteristics with NF and SF were carried out using chi-square statistics. To assess the relationship between disease status and gender, age, and place of residence crude analysis was conducted. Three outcomes were modeled using multivariate logistic regression to adjust for confounding. All statistical significance was tested at a significance level of 0.05 level.

### 2.3. Ethical Aspects

Ethical clearance for the study was obtained from the Ethics Review Committee of Faculty of Medicine, University of Colombo, Sri Lanka.

## 3. Results

There were 185 and 415 cases from North and South, respectively. An additional group of cases presented from Central (n=20), Western (n=104), and Northwestern (n=53) foci. Analysis pertaining to main foci is first given below.

### 3.1. Spatiotemporal Distribution of Cases

**Figure 2:**
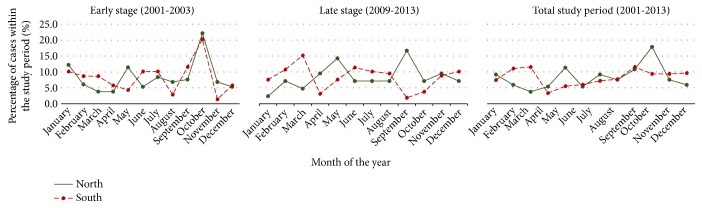
Trends in seasonal variation of case presentation in Northern and Southern study sites. Both North and South foci independently demonstrated a nearly similar and biannual seasonal variation pattern.

Majority of patients presented from North focus and South focus acquired the infection also from their same resident areas (data not shown). A similar pattern had been shown during the early stage of the epidemic also. Both foci independently also demonstrated a nearly similar and biannual seasonal variation pattern (Figure 2). Two peaks were observed during the early and latter parts of the year. Pattern was similar when the early stages of the epidemic were examined (Figure 2).

### 3.2. Variation in Sociodemographic Features in Two Main Foci

Majority were males in both foci with a markedly less male preponderance at SF (82.6% in North vs 58.8% in South). All age groups were almost equally affected in South while young adult age group (21-40 years, 74.6%) was mainly affected in North. Similar age and gender compositions were observed during the early and late stages as well (Table 1).

### 3.3. Variation in Clinical Profiles in Two Main Foci

Only a minority (<0.05%) of the studied cases at both sites had any of the studied systemic features and therefore are not comparable (fever, loss of appetite, loss of weight, pallor, jaundice, and hepatosplenomegaly).

According to the primary crosstabulation of data, majority of lesions at main transmission sites (NF, SF) presented as typical primary lesions (97.8% in North and 96.9% in South) on exposed body areas (89.7 in North and 96.9 % in South). Though majority remained single in both sites, SF reported more single lesions as compared to NF (73.5% in North and 92.5% in South). This feature was observed during early stage as well (Table 1). However, lesions in SF had a shorter mean (SD) duration of 5.62 (6.3) months as compared to 10.07 (25.3) months in NF. Longer duration observed in North was significant.. Lesions presented at NF had a longer duration during early stage as well (data not shown).

Lesions reported from Northern focus were more likely to be nonulcerative, small (≤ 2cm), rounded, less erythematous, and even edged as compared to those of SF (Table 2). Nodular lesions in NF showing less surface squamation and ulcers in NF were less likely to be moist when compared to similar stages of lesions in SF (Table 2). Surrounding skin of lesions also showed less scaling, inflammation, and altered pigmentation in NF as compared to those of SF (Table 2). All these differences except for skin pigmentation (for which P values were not acceptable due to low sample size) were statistically significant (Table 2).

### 3.4. Variations within Southern Focus

Minority of Southern Sri Lankan resident patients served as soldiers in Northern Sri Lanka and stayed in North for most of the time (n=19) (Table 3). Civilian group (who mainly stayed in Southern Sri Lanka) presented a clinical picture more consistent with SF profile while soldiers demonstrated a clinical profile more closer to what is reported in NF (Table 3). Proportion of single lesions, >2 cm sized lesions, ulcerated lesions, and lesion itchiness were markedly less among soldiers who probably acquired infection from North than those reported among civilians who probably were infected while in South. Soldiers also demonstrated less skin inflammation and altered pigmentation patterns though statistical significance could not be demonstrated probably due to low case numbers.

### 3.5. Examination of Potential Underlying Factors (Age, Gender, or Spatial Location)

Age, gender, and the spatial location were examined by logistic regression modelling for the potential role as underlying reasons for clinical differences observed between two main disease foci. Ulcerative stages were 3 times more likely to occur in SF as compared to NF (Table 4). Multiplication was more likely to occur in North while enlargement was more likely to occur in South (Table 3). Age or sex dependent variations in ulceration, multiplication, or enlargement of lesions were very unlikely while only region based differences were more apparent (Table 4).

### 3.6. Other Transmission Foci

**Figure 3:**
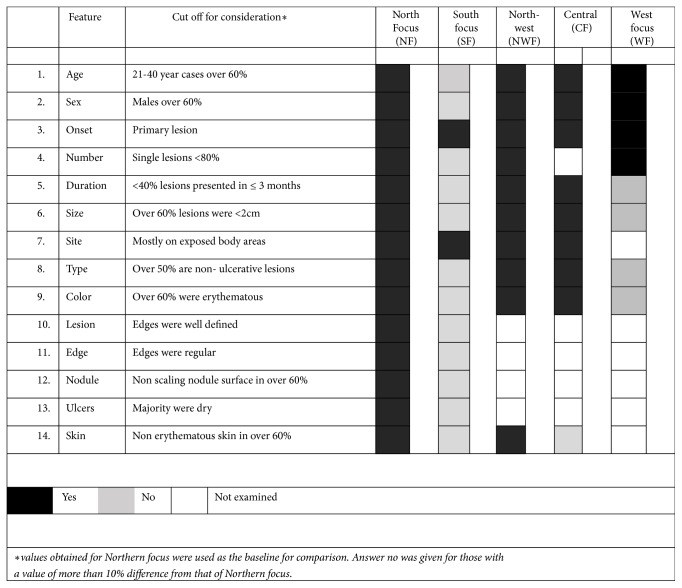
Graphical presentation of region based clinical variation within the country. Clinical profiles observed in SF were different from NF. NWF and CF showed close relationship with the profile seen in NF while the WF showed a mixed picture.

There were small number of patients presented from WF (n=118), CF (n=20), and NWF (n=53). Further analysis of their detailed clinical profile showed that features observed in South were different from those observed in North (Figure 3). However, clinical profile in Northwest and Central foci showed close relationship with the profile seen in North while the Western focus showed a mixed picture (Figure 3).

## 4. Discussion

Epidemiological evidence for presence of two main disease prevalent areas within the island was recently shown [16]. Different sociodemographic and clinical characteristics relating to these two major leishmaniasis prevalent areas were identified in the current analysis. Many findings of this study pointed towards long-term existence of independent and different disease transmission sites in North and South.

Both Northern and Southern Sri Lankan study areas largely share the features of dry zones in Sri Lanka. Presence of a seasonal pattern of case presentation in both regions approximately coincides with the local monsoonal rainfall patterns. Due to slight deviations of climatic factors observed at present, this variation could have been less prominent. Observation of same clinical and seasonal patterns in each focus since long (during early stage) further favours a long established nature of disease transmission in the two areas. Furthermore, Phlebotomine sand flies are widely prevalent in Sri Lanka since long including Northern, Southern, and Central Sri Lanka [29–31]. In spite of the general belief of only morphospecies B as the potential vector, both morphospecies A and B have been identified as potential vectors in Sri Lanka [31]. Such differences in vector populations in the different locations would have played a role in independent disease propagation and ultimate phenotypic differences too in these areas through facilitation of genetic structure variations in respective parasite populations. Prevalence of the different sand fly populations in these areas is not yet fully known. Currently available evidence with regard to the local animal reservoir hosts is too premature to support or contradict any finding [32].

Both study sites showed a male preponderance probably due to behavioural factors. Patient population in NF consisted of more males from armed forces. This may have slightly exaggerated the age and gender proportions within the true picture. Infection among young adult male population in NF further supports zoonotic transmission in this area while wider age distribution and increasing number of females in SF favour peridomestic transmission in South, a phenomenon demonstrated in previous studies also [17, 18]. This trend remained the same over the study period, further pointing towards established and stable disease transmission in the two areas. Subsequent peridomestication of initially zoonotic transmission cycles of leishmaniasis has been reported in the world.

**Figure 4:**
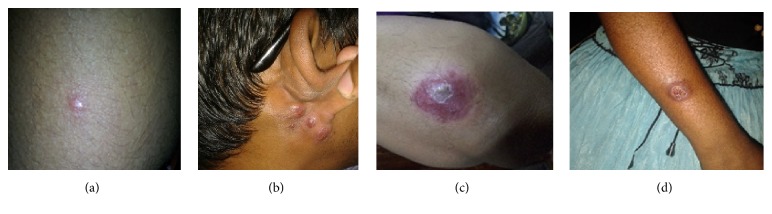
Different stages of a skin lesion in leishmaniasis, (a) early papular lesions, (b) multiple enlarging nodules, (c) an ulcerating nodule, and (d) a chronic ulcer.

Dermotropic nature of CL in Sri Lanka was demonstrated at many occasions. In both study sites there was minimal presence of systemic features. Skin lesions in both sites generally followed the generally known trend for occurrence of single and primary lesion over an exposed body area. Classical developmental stages of a leishmanial skin infection (Figure 4) were observed in all geographical locations. However, skin lesions reported in South were more likely to show rapid enlargement and ulceration while leishmanial skin lesions in North were more likely to remain small, nonulcerative, and multiplied slowly. Lesions at SF were more inflammatory as indicated by higher proportion of lesions showing surface scaling during nodular stages, lesion, and skin inflammatory changes as compared to those reported at NF which were less inflammatory. Rapid progression and inflammatory and associated features observed in South may indicate a more pronounced host reaction and/or pronounced immunity levels in South as the underlying reasons. Presence of higher proportions of lesions having nodular surface scaling, inflammatory changes in the lesion and surrounding skin, loss of regularity, and defined nature of lesions seen in Southern study area as compared to North further confirm this possibility. Pronounced immunity levels in patients from South may have resulted from early disease establishment in this area. First case of local transmission was also reported from South [6]. During the early stages of the outbreak majority of cases were from Northern Sri Lanka [7]. However, based on lesion patterns, and also considering the peridomestic and stable nature of transmission in South, the southern focus appears to be older.

It can also be argued that the parasite strains found in North coevolved with host in a better manner and became successful survivors within the host resulting in minimal host reaction. Favouring the latter possibility, soldiers from Southern Sri Lanka who most probably acquired infection during their work in Northern parts of the country demonstrated a basic clinical pattern consistent with the one described for Northern focus. These observed differences were not well marked to the level observed when North and South study populations were compared, probably due to low case numbers analysed and some soldiers acquiring infection from their resident areas in South.

These characteristics provide useful clinical clues for presence of different parasite strains in North and in South. This further favours independent progression of two transmission cycles which are most likely to be parasite dependent. Meanwhile, Northern focus has shown a slight trend towards a spreading/deviating spatial pattern while the Southern location seems to remain rather constant further favouring a parasite strain variation, rather than other contributing factors. These differences in each site may also indicate a difference in the rate of disease progression at two sites with Northern focus showing a relatively slow lesion progression while Southern focus lesions were more prone to show rapid ulceration and enlargement. During the multivariate analysis, differences in spatial locations rather than age or gender based differences were identified to be the underlying determinants for the two different clinical patterns seen in North and South. Consistency of each clinical pattern in its spatial location further favours the hypothesis for long-term existence and independent nature of disease transmission in these areas.

This study further identified that other case reporting areas in North, Western, and Central foci also possess many characteristics similar to those of North focus. They are likely to have acquired the spreading infection from a primary focus in any of these areas in Northern Sri Lanka. It is difficult to exactly point to a single location. Western focus which is not a high leishmaniasis prevalent region that includes the country's commercial capital demonstrated a mixed picture. Although leishmaniasis in WF is not known to be highly prevalent, the region is a highly populated and an urban area in the country with a large work populations moving in and out daily. Reported cases in WF were probably due to disease establishment in WF due to increased patient travel between WF and other different disease prevalent areas in the country. Sand fly vectors that are widely prevalent in the country including WF may have subsequently facilitated onward propagation of parasites in these areas.

Long-term disease existence may have probably allowed the transmission patterns to coevolve with the environmental conditions resulting in eventual establishment of leishmaniasis transmission cycles. Vigorous insecticide spraying was practised in Sri Lanka until 1960s as an antimalarial measure. Along with the reduction of malaria cases in the island, spraying activities were also reduced. These may have resulted in increased sand fly populations which in turn resulted in a gradual and silent increase in the infected human reservoirs (with or without animal reservoirs). More jungle associated military operations probably brought human reservoirs in close contact with scrub jungles in North during the past 2 decades favouring peridomestication of initially sylvatic cycles. Active measures taken to raise community and professional awareness following the detection of the spot case in year 2001 also may have played a role in the recent detection of this recently increased but long existing infection.

Even though it is likely that most patients probably acquired the infection from the same resident region (North or South) except for the soldiers who spent most of their time in Northern Sri Lanka, case prevalence or disease transmission intensities cannot be expected to be homogenous within any region. Presence of nonhomogenous disease transmission within each region is likely and already known. Fine scaled studies are likely to reveal further interesting and useful information. Parasite strain variation is likely to show considerable differences in the two main regions in spite of the previous finding of* L. donovani*. Lesions observed in North and South seem to resemble* L. tropica* or* L. major* in other different endemic geographical locations, though* L. donovani* is already known to cause CL in this focus. Strain variation studies may reveal further information. Identification of spatiotemporal distribution of cases in affected areas, study of spread of disease transmission hotspots over the time, understanding the related ecological and climatic factors, study of sociodemographic variations, region based descriptions of species, prevalence, behaviours, and insecticide susceptibility patterns of sand fly populations are important in interrupting leishmaniasis transmission in these areas. In addition, study of drug sensitivity patterns in the two regions may reveal useful information.

## 5. Conclusions

Regional variation in clinical and sociodemographic characteristics between North and South was identified. Consistency of the identified patterns in each focus, independency in disease transmission in each focus, and continuation of identified variation between two sites were demonstrated in this study. The findings of this study suggest that both NF and SF were preexisting foci, and not newly emerging, and that a continued transmission seemed to contribute to the increasingly higher prevalence observed in recent years. Findings further supported a parasitic aetiology for the clinical variation. Northwestern and Central foci are likely to be the result of expanding or shifting Northern disease transmission focus. Southern focus, which is probably the older focus out of the two, seems to be clearly different from other disease foci and remains confined to the same area within the island.

Sequelae of* L. donovani *skin infection are not yet fully understood. Presence of multiple strains within the group of local* L. donovani* indicates more future complexities which cannot be predicted completely or accurately at this moment. It is always possible that the local variants further undergo genetic changes which could result in unfavourable clinicoepidemiological outcomes as well. Recently detected VL and mucosal leishmaniasis, changing clinical profiles, an atypical entity within the CL profile, and poor treatment response patterns in the local setting already attest to this possibility. Studies in all these areas, proper description of the ongoing disease outbreak, underlying aetiologies, evidence based disease control strategies, allocation of adequate resources, and persistence in work are essential.

## Figures and Tables

**Table 1:** Trends in socio demographic and clinical features in Northern and Southern study sites over the epidemic in all three stages (early and late stages).

	Early stage (2001-2003)				Late stage (2009-2014)				Total period (2001-2013)			
	North		South		North		South		North		South	
	Count	(%)	Count	(%)	Count	(%)	Count	(%)	Count	(%)	Count	(%)
Age (years)												
Up to 20	6	(4.6)	17	(24.6)	7	(16.6)	46	(29.1)	19	(10.3)	112	(27.0)
21-40	113	(86.2)	24	(34.8)	22	(52.4)	55	(34.8)	138	(74.6)	141	(34.0)
Over 40	12	(9.2)	28	(40.6)	13	(31.0)	57	(36.1)	28	(15.1)	162	(39.0)
Total	131	(100.0)	69	(100.0)	42	(100.0)	158	(100.0)	185	(100.0)	415	(100.0)
Sex												
Male	124	(94.7)	41	(59.4)	20	(48.8)	88	(55.7)	152	(82.6)	244	(58.8)
Female	7	(5.3)	28	(40.6)	21	(51.2)	70	(44.3)	32	(17.4)	171	(41.2)
Total	131	(100.0)	69	(100.0)	41	100.0)	158	(100.0)	184	(100.0)	415	(100.0)
Number												
Single	93	(71.0)	54	(78.3)	35	(83.3)	149	(94.3)	136	(73.5)	384	(92.5)
Multiple	38	(29.0)	15	(21.7)	7	(16.7)	9	(5.7)	49	(26.5)	31	(7.5)
Total	131	(100.0)	69	(100.0)	42	(100.0)	158	(100.0)	185	(100.0)	415	(100.0)
Site												
Exposed∗	118	(90.1)	62	(89.9)	39	(92.9)	145	(91.8)	166	(89.7)	376	(90.6)
Not	13	(9.9)	7	(10.1)	3	(7.1)	13	(8.2)	19	(10.3)	39	(9.4)
Total	131	(100.0)	69	(100.0)	42	(100.0)	158	(100.0)	185	(100.0)	415	(100.0)
Onset primary lesion												
yes	129	(98.5)	68	(98.6)	41	(97.6)	149	(94.3)	181	(97.8)	402	(96.9)
No	2	(1.5)	1	(1.4)	1	(2.4)	9	(5.7)	1	(2.2)	13	(3.1)
Total	131	(100.0)	69	(100.0)	42	(100.0)	158	(100.0)	185	(100.0)	415	(100.0)

*∗*Upper and lower limbs, head, face and neck areas.

**Table 2:** Comparison of clinical features between main disease transmission foci.

*Lesion features *	*North (NF)*		*South (SF)*		*P value*
	Count	(% )	Count	(%)	
Type*∗*					
NUT	128	(69.2)	193	(46.5)	<0.0001
UT	57	(30.8)	222	(53.5)	
Total	185	(100.0)	415	(100.0)	
Size					
≤2 cm	126	(68.1)	220	(53.0)	<0.0001
>2 cm	59	(31.9)	195	(47.0)	
	185	(100.0)	415	(100.0)	
Shape					
Round	135	(73.0)	198	(47.7)	<0.0001
Oval	22	(11.9)	171	(41.2)	
Irregular	28	(15.1)	46	(11.1)	
Total	185	(100.0)	415	(100.0)	
Edge*∗∗*					
Regular	124	(68.5)	163	(53.2)	<0.0001
Not	57	(31.5)	143	(46.7)	
Total	181	(100.0)	306	(100.0)	
Surface scaling in nodules*∗∗*					
Observed	40	(28.6)	81	(59.5)	<0.0001
Not	100	(71.4)	55	(40.5)	
Total	140	(100.0)	136	(100.0)	
Dry or moist ulcer*∗∗*					
Dry	40	(75.5)	74	(44.6)	<0.0001
moist	5	(9.4)	68	(41.0)	
Uncertain	8	(15.1)	24	(14.5)	
Total	53	(100.0)	166	(100.0)	
*Surrounding skin feature* ^$^					
Scaling*∗∗*^$^	39	(23.5)	65	(49.2)	<0.0001
Inflammation*∗∗*^$^	26	(16.5)	43	(39.1)	<0.0001
Altered pigmentation*∗∗*^$^	34	(79.1)	11	(61.1)	NC

NUT: non ulcerative type of lesions, UT: ulcerative type of lesions, *∗∗* missing data excluded, ^$^ only positive categories were shown.

**Table 3:** Comparison of univariate and multivariate analysis of disease patterns.

	Disease pattern	Crude odds ratio and CI	Adjusted OR and CI
*Model 1 for ulcerated lesions (n=178)*	*Sex*		
	Female	0.713 (0.506- 1.0)	*0.538 (0.37-0.78)*
	Male	ref	ref
	*Age (in years)*		
	Over 40	1.14 (0.73-1.77)	1.13 (0.72-1.79)
	21-40	0.85 (0.56-1.29)	1.02 (0.64-1.62)
	Up to 20 years	ref	ref
	*Place of residence*		
	South	*2.583 (1.79-3.72)*	*3.024 (2.01-4.54)*
	North	ref	ref

*Model 2 for multiple lesions (n=80)*	*Sex*		
	Female	*0.41 (0.22-0.73)*	0.69 (0.36- 1.32)
	Male	ref	ref
	*Age (in years)*		
	Over 40	1.51 (0.63-3.61)	1.47 (0.61-3.56)-
	21-40	*3.77 (1.74-8.18)*	2.19 (0.91-4.87)
	Up to 20 years	ref	ref
	*Place of residence*		
	South	*0.22 (0.14-0.37)*	*0.29 (0.17-0.49)*
	North	ref	ref

*Model 3 for enlarged lesions (n=346)*	*Sex*		
	Female	1.104(0.78-1.554)	1.07 (0.74-1.55)
	Male	ref	ref
	*Age (in years)*		
	Over 40	*0.48 (0.30-0.75)*	*0.47 (0.29-0.75)*
	21-40	0.87 (0.56-1.34)	0.72 (0.45-1.15)
	Up to 20 years	ref	ref
	*Place of residence*		
	South	*0.53 (0.37-0.76)*	*0.53 (0.36-0.79)*
	North	ref	ref

**Table 4:** Comparison of clinical profile in soldiers and civilians from Southern Sri Lanka (n=379).

	Clinical features of lesion	Military (LPIA*∗∗* north)		Civilian (LPIA*∗∗* South)	
		Count	(%)	Count	(%)
Number of lesions*∗*	Single	11	(57.9)	341	(94.7)
	Multiple	8	(42.1)	19	(5.3)
	Total	19	(100.0)	360	(100.0)

Duration*∗*	<6months	14	(73.7)	282	(78.3)
	>6 months	5	(26.3)	78	(21.7)
	Total	19	(100.0)	360	(100.0)

Lesion size*∗*	≤2 cm	14	(73.7)	183	(50.8)
	>2 cm	5	(26.3)	177	(49.2)
	Total	19	(100.0)	360	(100.0)

Lesion type	Non-ulcerated	9	(64.3)	24	(48.0)
	Ulcerated	5	(35.7)	26	(52.0)
	Total	14	(100.0)	50	(100.0)

Lesion site	Distal limbs	10	(71.4)	21	(42.0)
	Proximal limbs	0	(0.0)	1	(2.0)
	Trunk	1	(7.1)	5	(10.0)
	Head and Neck	3	(21.4)	23	(46.0)
	Total	14	(100.0)	50	(100.0)

Itchiness*∗*	From beginning	1	(5.3)	18	(21.7)
	Never	18	(94.7)	65	(78.3)
	Total	19	(100.0)	83	(100.0)

Skin scaling*∗*	Yes	7	(36.8)	53	(50.0)
	No	12	(63.2)	53	(50.0)
	Total	19	(100.0)	106	(100.0)

Skin inflammation*∗*	Yes	2	(11.8)	26	(33.3)
	No	15	(88.2)	52	(66.7)
	Total	17	(100.0)	78	(100.0)

Skin pigmentation*∗*	Yes	6	(75.0)	5	(55.6)
	No	2	(25.0)	4	(44.4)
	Total	8	(100.0)	9	(100.0)

*∗*missing data were excluded, *∗∗* LPIA: likely place of infections acquisition

## Data Availability

Data has not been made available as it was not part of the ethics application and due to patient confidentiality.
